# Serum matrix metalloproteinase-7 levels in infants with cholestasis and biliary atresia

**DOI:** 10.1186/s12887-022-03409-9

**Published:** 2022-06-18

**Authors:** Pejman Rohani, Seyyed Bahador Mirrahimi, Haleh Bashirirad, Parisa Rahmani, Niyoosha Kamran, Hosein Alimadadi, Mahmoud Hajipour, Mohammad Hassan Sohouli

**Affiliations:** 1 Pediatric Gastroenterology and Hepatology Research Center, Pediatrics Centre of Excellence, Children’s Medical Center, Tehran University of Medical Sciences, Tehran, Iran; 2 Department of Clinical Nutrition and Dietetics, Faculty of Nutrition and Food Technology, Shahid Beheshti University of Medical Sciences, Tehran, Iran; 3 Department of Pediatrics, School of Medicine Childrens Medical Center, Tehran University of Medical Sciences, Tehran, Iran

**Keywords:** MMP7, GGT, Biliary atresia, Alkaline phosphatase

## Abstract

**Background:**

The aim of this study was to evaluate the serum level of matrix metalloproteinase 7 (MMP7) in infants with cholestasis and the diagnostic values of this biomarker to differentiate biliary atresia (BA) from other causes of cholestasis.

**Methods:**

This multi-center study is conducted during 2 years in Mofid children’s hospital and Children’s Medical Center, Pediatrics Center of Excellence Tehran, Iran. 54 infants with cholestasis were enrolled in this study with a control group consists of 41 healthy infants with the same age. Serum samples were taken from all these patients to assess serum levels of MMP7, Gamma-glutamyl Transferase (GGT). For each biomarker, we calculated the sensitivity and specificity and other statistical characteristics.

**Results:**

There were 89 subjects, 22 patients with BA, 32 patients with non-BA cholestasis and 41 subjects as control group. The mean serum MMP7 levels in BA, non-BA cholestasis and control group was 15.91 ng/ml ± 6.64, 4.73 ng/ml ± 2.59 and 0.49 ng/ml ± 0.33, respectively. The best cut-off point is calculated 7.8 ng/ml for MMP7 and 434.5 U/L for GGT. The area under curve (AUC) for these two markers are 0.988 ± 0.008 and 0.854 ± 0.052, respectively. The sensitivity and specificity of MMP7 to differentiate biliary atresia from nonbiliary atresia cholestasis in our study was 95.5% and 94.5%, respectively. The sensitivity and specificity of GGT was 77.3% and 77.8%, respectively. These results show that the MMP7 has more sensitivity and specificity in differentiation.

**Conclusion:**

MMP7 demonstrated good accuracy to differentiate biliary atresia from other causes of cholestasis.

## Introduction

Biliary atresia (BA) is a progressive fibroinflammatory disease of the internal and external hepatic ducts that predisposes to liver transplantation in children [1]. The disease presents with jaundice, hepatomegaly, and acholic stools, and these symptoms are similar to those of other cholestatic liver diseases [2]. Earlier diagnosis of this disease is important because earlier treatment with Kasai procedure is associated with the best chances of delaying and avoiding need for liver transplant [3]. However, there is currently no certain non-invasive diagnostic modality to diagnose BA and differentiate it from other causes of cholestasis [4]. Routine biochemical tests are currently used to assess neonates with cholestasis for degree of cholestasis, hepatocellular damage, and hepatocyte function [4]. Abnormalities are also diagnosed by ultrasound, traditional Hepatobiliary sequence scintigraphy, endoscopic retrograde cholangiopancreatography and also liver biopsy [5].

Matrix metalloproteinases are a group of enzymes that separate the extracellular matrix from zinc compounds [6]. These proteinases are inactive at first but become active after release from the cell [6]. Ischemia and reperfusion of hepatocytes release the matrix of metalloproteinases, and chronic release of these enzymes may destroys liver tissue and may be considered a potential mechanism [7]. Sinusoidal endothelial cells are the main secretory cells, although satellite and Kupffer cells are also involved in its production [8–10].

Previous studies have reported an increase intrahepatic matrix metalloproteinase-7 (MMP-7) expression level in infants with biliary atresia [5, 11]. The results of a study showed that infants with BA had a significantly higher serum MMP-7 level than that of non–BA infants with cholestasis of equivalent age [3]. Also, receiver operating characteristic analysis showed that a serum MMP-7 level of > 1.43 ng/mL was predictive of BA in infants with cholestasis (diagnostic accuracy, 88%) [3]. With these promising results it can be a useful biomarker in differentiating biliary atresia from other causes of cholestasis, but it is still not used as a routine test [12]. Hepatic fibrosis occurs earlier and faster in infants with BA than other causes of cholestasis and MMP7 is involved in the development of liver fibrosis, therefore inhibitors of this factor may be useful in preventing liver fibrosis [13]. Studies have also reported a strong association between serum levels of this enzyme and the severity of liver fibrosis [7, 10, 14].

The aim of this study was to evaluate the serum level of matrix metalloproteinase 7 in infants with cholestasis and the diagnostic values of this biomarker to differentiate BA from other causes of cholestasis. Also, considering that before MMP7, Gamma-glutamyl transferase (GGT) was used as a suitable biomarker to differentiate these two diagnoses (BA than other causes of cholestasis), we compared specificity & sensitivity and diagnostic accuracy of these two biomarkers.

## Methods and materials

### Study design and population

A multi-center study (case control study) was performed during 2 years (October 2018 to September 2020) in Mofid children’s hospital and Children’s Medical Center, Pediatrics Center of Excellence Tehran, Iran. All neonates and infants with cholestasis referred to these medical pediatrics center were included in the study (54 patients with cholestasis were enrolled). All patients underwent exploratory laparotomy and were divided into two categories of biliary atresia and other causes of cholestasis. To confirm the diagnosis of neonates and infants BA, intraoperative cholangiography and examination of liver biopsies were used. Due to the fact that contrast injection was difficult in these patients and the gallbladder of some of these people was anatomically completely atrophic, BA was approved once and diagnosed by a physician. Otherwise, before the final diagnosis of the disease, the anatomical tissue of the intrahepatic bile ducts was examined and evaluated using cholangiography. Patent biliary tree observation by intraoperative cholangiography was also used to confirm non-BA cholestasis neonates and infants. The distinction between BA and other causes of cholestasis in neonates during surgery is cholangiography, which was performed before cassation surgery. Also, to evaluate two target biomarkers (MMP7 and GGT) in a healthy population to detect baseline values, 41 healthy infants under 6 months of age were added to the study as a control group. Finally, the study data were statistically analyzed and the statistical characteristics of these two biomarkers were extracted to differentiate BA from other causes of cholestasis. In tables, "Age (day)" means the age of patients at the beginning of the study and sampling (participation in the study) and “Age at diagnosis (day)" means the age at which a person is diagnosed with the disease, both before and after the study. The current weight of the participants is in fact when the individuals enter the study who are interviewed and the necessary data are taken from them. Also, the infants of the healthy group were infants who were either born in the same hospital at the time of the study or were infants with various causes such as eye, ear and bone problems (without any liver or biliary problems and rejection of other causes related to Disease) were referred to the same hospital at the time of sampling. The Ethical Committee of the Faculty of Medicine, Tehran University of Medical Sciences approved the study (Ethical number: IR.TUMS.MEDICINE.REC.1400.1529). There was no additional intervention in the process of this study and no physical and spiritual burden were borne by the examined patients. All patients had informed consent about participating in the study and there were no harm or excessive charge on patients.

### Biochemical studies

Upon arrival, blood samples were taken for the tests evaluated in this study. After clotting in the environment, the serum was isolated as soon as possible by centrifugation and kept at -70° C until sent to the laboratory for testing. MMP-7 levels were measured by using an enzyme-linked immunosorbent assay (ELISA; DuoSet, R&D Systems, Inc, Minneapolis, Minnesota) and monoclonal MMP-7 antibody, clone 141-7B2 (Merck Millipore, Merck KGaA, Darmstadt, Germany). Serum levels of GGT, Alkaline phosphatase (ALP), alanine aminotransferase (ALT), aspartate aminotransferase (AST), total bilirubin, direct bilirubin, and CBC were determined as part of the evaluation for all cholestatic patients. Liver enzymes (ALT, AST, GGT, and ALP) concentration were measured using Pars Azmon Company kit (Pars Azmon, Tehran, Iran) and enzymatic colorimetric method.

### Statistical analysis

After proving the normality of the distribution of the studied variables by Kolmogorov–Smirnov test, one-way analysis of variance (ANOVA) used to compare quantitative variables between the three groups and T-Test between the two groups. Chi-square or fisher exact statistical test was also used for qualitative variables. Correlations were calculated using Spearman's rank correlation to examine the correlation between age at diagnosis of disease with MMP-7. To measure the detection power of biomarkers in predicting the final result, we used the statistical indicators of sensitivity, specificity, positive and negative predictive value, and the Yuden index. We also plotted the ROC diagrams separately for markers to obtain the optimal point and a suitable cut-off point. All analyzes were performed by SPSS 25.0 statistical software and *P*-value less than 0.05 was considered statistically significant.

## Results

We enrolled 54 neonates and infants less than 6 months of age with cholestasis, 22 subjects with BA and 32 with non-BA cholestasis and 41 subjects as control group with median age of 50.09 ± 26.38, 64.29 ± 35.38 and 79.93 ± 63.14 day, respectively. As shown in Table 1, of 22 patients with final diagnosis of BA (based on pathology reports), 12 (54.5%) were boys and 10 (45.4%) were girls. In this regard there was 19 boys and 22 girls in healthy individuals and 17 boys and 15 girls in patients with non-BA cholestasis (*P*-value = 0.773). The mean age and weight of patients were not statistically different between the two groups, but age at diagnosis and birth weight were significant different among patients diagnosed with BA and non-BA cholestasis (*p*-value = 0.03). Patients with BA were heavier at birth (*p*-value = 0.027).

**Table 1:** Demographic information of patients by study groups

		**Diagnosis**			
		**Biliary atresia, *N* = 22**	**Healthy, *N* = 41**	**non-BA cholestasis, *N* = 32**	***P*-value**^a^
**Gender**	Male	12 (54.5%)	19 (46.3%)	17 (53.1%)	0.773
	Female	10 (45.5%)	22 (53.7%)	15 (46.9%)	
**Age of participant (day)**		50.09 ± 26.38	79.93 ± 63.14	64.29 ± 35.38	0.062
**Age at diagnostic laparotomy/KPE (day)**		21.68 ± 10.87	___	32.61 ± 20.29	**0.03**
**Birth weight (gr)**		3060.53 ± 254.7	___	2798.28 ± 454.96	**0.027**
**Current weight (gr)**		4227.22 ± 1044.02	4892.68 ± 1666.49	4113 ± 1086.51	0.078

^a^ Obtained from ANOVA for continuous variables and Chi-square of independence for Categorical variables. Values are mean ± SD (95% CI) or n (%)

A significance level of 0.05 was considered (*P*value < 0.05)

The final diagnosis for patients non-BA cholestasis included cytomegalovirus (CMV) hepatitis (*n* = 8), Alagille syndrome (*n* = 5), citrin deficiency (*n* = 1), progressive familial intrahepatic cholestasis (*n* = 3), parenteral nutrition–associated cholestasis (*n* = 1), intrahepatic bile duct dysplasia (*n* = 1), congenital absence of a gallbladder (*n* = 1), gallbladder duplication (*n* = 1), neonatal sclerosing cholangitis (*n *= 1), and idiopathic cholestasis (*n* = 10). According to the classification of anatomical types of biliary atresia (BA), 15 (68.18%) infants were type 4 patients, 4 (18.18%) infants were type 3, 2 (9.09%)infants were type 2, and one (4.54%)infant was type 1, and all were congenital biliary atresia [15].

As shown in Table 2, GGT and MMP7 levels between these three study groups were significantly different (*P*-value < 0.001). Also, the difference in mean ALP between the BA and non-BA cholestasis group was significant (*P*-value = 0.004). But differences between serum AST, ALT, Bilirubin direct, bilirubin indirect, WBC, hemoglobin, PLT and MCV were not statistically significant. The mean serum MMP7 levels in BA, non-BA cholestasis and control group was 15.91 ± 6.64, 4.73 ± 2.59 and 0.49 ± 0.33, respectively. The mean serum GGT levels in BA, non-BA cholestasis and control group was 551.73 ± 208.17, 274.04 ± 163.43 and 149.71 ± 58.53, respectively. The difference in serum levels of MMP-7 and GGT between BA and non-BA cholestasis were 11.18 and 277.69, respectively. The mean serum ALP levels in BA and non-BA cholestasis group was 1765.1 ± 830.55 and 1042.21 ± 787.29, respectively.

**Table 2:** Mean levels of MMP7, GGT and other markers among the three study groups

	**Diagnosis**			
	**Biliary atresia**	**healthy**	**non-BA cholestasis**	***P*-value**^a^
**Bilirubin total (mg/dl)**	14.48 ± 4.84	___	13.88 ± 4.89	0.669
**Bilirubin direct (mg/dl)**	6.89 ± 3.54	___	5.72 ± 2.75	0.187
**AST (U/L)**	202.75 ± 89.14	___	285.77 ± 197.63	0.085
**ALT(U/L)**	119.1 ± 58.78	___	173 ± 120.58	0.070
**ALP(U/L)**	1765.1 ± 830.55	___	1042.21 ± 787.29	**0.004**
**GGT(U/L)**	551.73 ± 208.17	149.71 ± 58.53	274.04 ± 163.43	< 0.001^*^
**Hemoglobin**	13.54 ± 1.54	___	13.1 ± 2.21	0.441
**MCV**	91.24 ± 2.74	___	119.14 ± 165.9	0.446
**WBC**	8311.9 ± 2903.23	___	7243.9 ± 3098.28	0.220
**PLT**	295,000 ± 130,000.38	___	323,433.33 ± 165,451.04	0.514
**MMP7 (ng/mL)**	15.91 ± 6.64	0.49 ± 0.33	4.73 ± 2.59	**< 0.001**^**#**^

*Abbreviation*: *AST* Aspartate aminotransferase, *ALT* Alanine aminotransferase, *ALP* Alkaline phosphatase, *GGT* Gamma-glutamyl transferase, *MCV* Mean corpuscular volume, *WBC* White blood cells, *PLT* Platelet (Thrombocyte) count, *MMP7* matrix Metalloproteinase-7

^a^ Obtained from ANOVA for continuous variables. Values are mean ± SD (95% CI) or n (%)

^*^ Significant differences between the Biliary atresia and healthy groups (*P*value < 0.001) and between the Biliary atresia and non-BA cholestasis groups (*P*value < 0.001) and also between the healthy and non-BA cholestasis groups (*P*value = 0.002) using Bonferroni test

^#^ Significant differences between the Biliary atresia and healthy groups (*P*value < 0.001**)** and between the Biliary atresia and non-BA cholestasis groups (*P*value < 0.001**)** and also between the healthy and non-BA cholestasis groups (*P*value < 0.001) using Bonferroni test

A significance level of 0.05 was considered (Pvalue < 0.05)

**Fig. 1:**
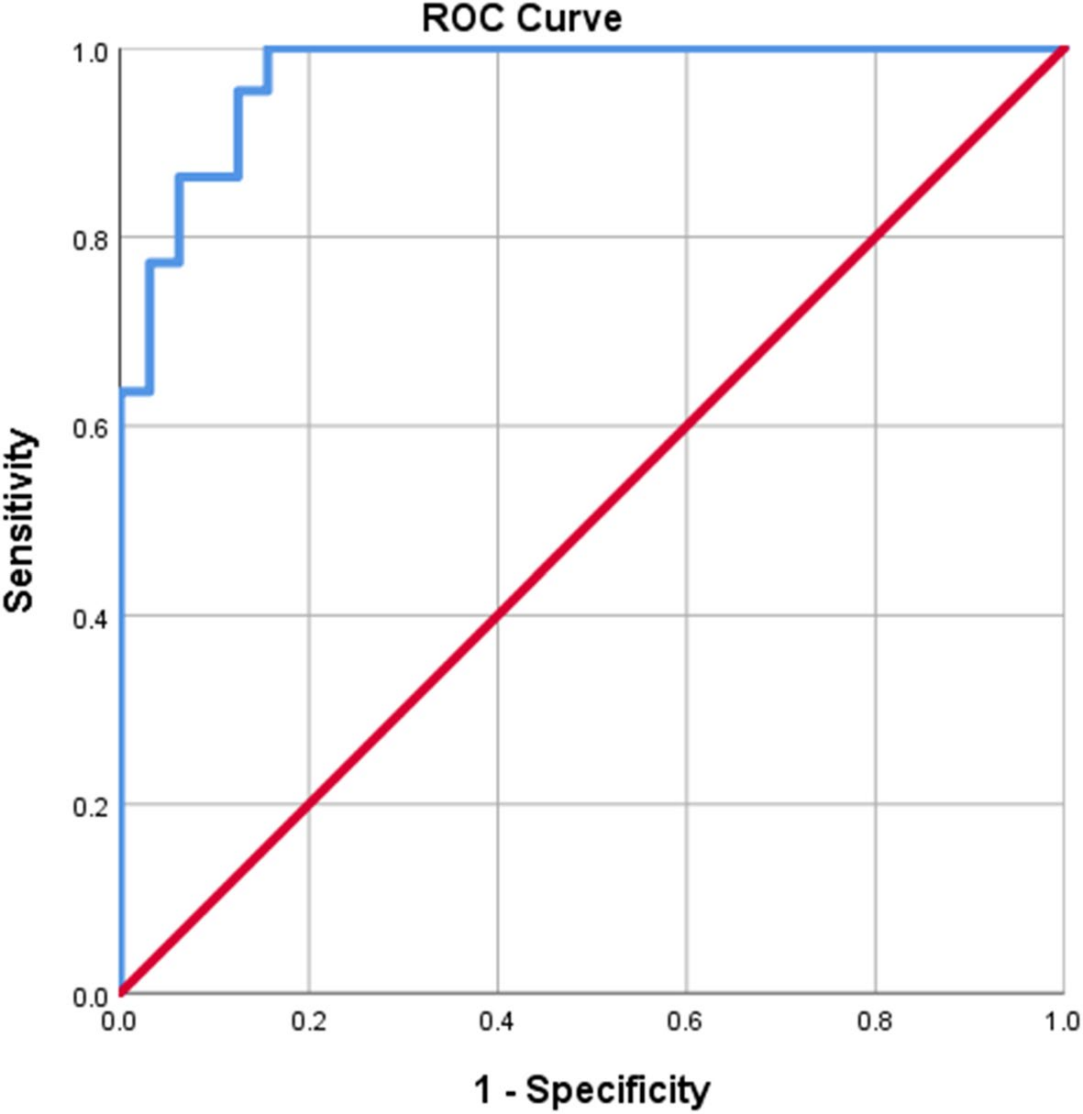
ROC curve for MMP7 marker

**Fig. 2:**
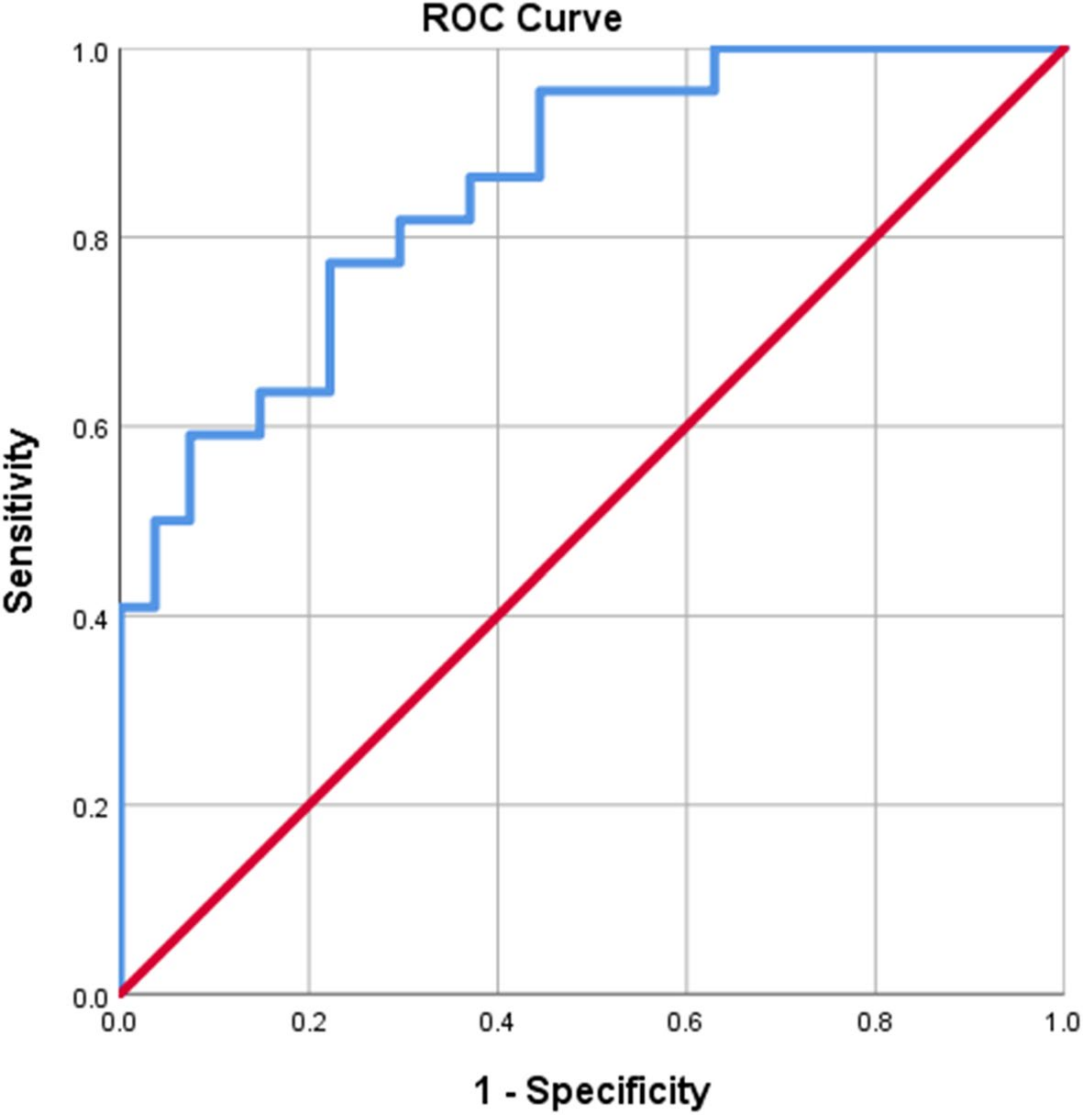
ROC curve for GGT

In order to determine the sensitivity and specificity and to calculate the best cutoff point and AUC for MMP-7 and GTT, receiver operating characteristic (ROC) curves were constructed for each marker separately, (Figs. 1 and 2). Based on this, the best cut-off points as well as the sensitivity and specificity for each of the marker values were calculated (Table 3). The best cut-off point is the number that has the highest accuracy in distinguishing BA patients from non-BA cholestasis. It’s calculated 7.8 ng/ml for MMP7 and 434.5 U/L for GGT. The area under curve (AUC) for these two markers are 0.988 ± 0.008 and 0.854 ± 0.052, respectively. The sensitivity and specificity of MMP7 to differentiate (diagnose) BA from non-BA cholestasis in our study was 95.5% and 94.5%, respectively. The sensitivity of MMP7 is 95.5. That is, out of 22 possible patients, 21 patients correctly predicted. This is assumed to be 7.8 ng/ml if the specified cutoff is set. The sensitivity and specificity of GGT was 77.3% and 77.8%, respectively. These results show that the MMP7 has more sensitivity and specificity in differentiation. Considering the AUC which is one of the best criteria for determining the predictive power, we reach the same conclusion.

**Table 3:** Statistical characteristics of MMP7 and GGT

	**AUC**	**95% CI**	***P*-value**	**Cutoff**	**Sensitivity (%)**	**Specificity (%)**	**Youden index**	**PPV**	**NPV**
MMP7 (ng/mL)	0.988 ± 0.008	0.972—1	** < 0.001**	7.8	95.5	94.5	0.844	81.82	93.75
GGT (U/L)	0.854 ± 0.052	0.751—0.956	** < 0.001**	434.5	77.3	77.8	0.551	71.27	77.8

*Abbreviation*: *GGT* Gamma-glutamyl transferase, *MMP7* matrix Metalloproteinase-7, *PPV* Positive predicted value, *NPV* Negative predicted value, *AUC* Area under ROC curve

Youden index = Sensitivity + Specificity

Table 4 examines the significance of the difference between MMP7 and GGT in men and women by study groups. As shown in this table, GGT is significantly higher among healthy boys than healthy girls (*p*-value = 0.001). Furthermore, both GGT and MMP7 were higher in boys with BA than girls (*p*-value = 0.012, *p*-value = 0.034, respectively).

**Table 4:** Comparison of MMP7 and GGT levels by patients’ gender

	**Total**			**Biliary atresia**			**non-BA cholestasis**			**Healthy**		
	**Male**	**Female**	***p*-value**	**Male**	**Female**	***p*-value**	**Male**	**Female**	***p*-value**	**Male**	**Female**	***P*-value**
**MMP7 (ng/mL)**	10.54 ± 8.59	7.82 ± 4.98	0.137	18.59 ± 7.23	12.68 ± 4.22	**0.034**	4.86 ± 3.21	4.58 ± 1.74	0.766	0.44 ± 0.04	0.54 ± 0.45	0.316
**GGT (U/L)**	428.56 ± 262.31	362.09 ± 182.14	0.063	649.17 ± 183.82	434.8 ± 178.8	**0.012**	252.07 ± 161.7	301.5 ± 168.44	0.446	180.74 ± 60.32	122.91 ± 42.24	**0.001**

Table 5 examines the differences between MMP7 and GGT in infants and neonates (≤ 28 days and > 28 days of life) by different study groups. GGT was significantly higher in healthy infants aged ≤ 28 days than in healthy infants aged > 28 days (*p*-value = 0.006). In other groups, although GGT levels were higher in infants older than 28 days than in infants younger than 28 days, no statistically significant difference was observed. Also, the correlation between age at the time of diagnosis and MMP-7 did not show a significant correlation (correlation coefficient = -0.225, *P*value = 0.137).

**Table 5:** Comparison of MMP7 and GGT levels by patients’ age

	**Total**			**Biliary atresia**			**non-BA cholestasis**			**Healthy**		
	≤ 28	> 28	*p*-value	≤ 28	> 28	*p*-value	≤ 28	> 28	*p*-value	≤ 28	> 28	*P*-value
**MMP7 (ng/mL)**	10.65 ± 5.82	8.97 ± 7.52	0.884	14.32 ± 4.04	16.5 ± 7.41	0.505	5.15 ± 2.67	4.67 ± 2.63	0.735	0.42 ± 0.02	0.52 ± 0.39	0.413
**GGT (U/L)**	381.4 ± 203.45	403.15 ± 238.58	0.923	476.83 ± 152.62	579.81 ± 223.21	0.313	238.25 ± 199.53	280.26 ± 160.82	0.644	190.27 ± 60.69	134.83 ± 51.01	**0.006**

Also, according to Table 6, after adjusting for possible confounders (ALP, age, birth weight, and MMP7 or GGT), we examined the effect of sex and age on serum concentrations of GGT and MMP-7 (*P*value > 0.05), but no significant relationship was observed.

**Table 6:** Relationship between age and gender with serum concentrations of GGT & MMP-7

	**MMP-7**			**GGT**		
	R^2^	B coefficient	*P*-value	R^2^	B coefficient	*P*-value*
**Gender **^**a**^	0.561	0.012	0.918	0.516	0.131	0.299
**Age **^**b**^	0.654	-0.159	0.182	0.5	0.51	0.693

^*^ *P*-value is less than 0.05 and significant

^a^ adjusted by ALP, age, birth weight, and MMP7 or GGT

^b^ adjusted by ALP, birth weight, and MMP7 or GGT

- Multivariate Linear regression analysis has been used

## Discussion

Biliary atresia (BA) is a rare cholangiopathy of infancy in which the bile ducts obliterate, leading to profound cholestasis and liver fibrosis through various factors such as impaired immune function and inflammatory factors [16, 17]. Identical clinical and biochemical symptoms and indistinguishable in these infants and other infants with cholestasis, leads to impaired timely diagnosis and appropriate treatment in these individuals. On the other hand, the prediction model of the classification decision based on direct bilirubin next to GGT and acholic stools for the diagnosis and differentiation of biliary atresia among other neonates with cholestasis also has a false negative rate of 11% [18]. Therefore, it is necessary to identify and discover a non-invasive method to differentiate BA among these infants. In this study, we examined MMP-7 levels as a predictive and differential biomarker among these infants. The results showed high sensitivity and specificity of this biomarker compared to GGT, which were reported as 95.5 and 94.5, respectively.

In a meta-analysis and systematic study in 2020 on 4 studies involving 593 neonates, it was reported that the mean sensitivity and specificity of MMP-7 for the detection of BA were 0.96 (95% CI: 0.93–0.98) 0.91 (95% CI: 0.85–0.95), respectively [11]. Also, the area below the reported curve was 0.97 (95% CI: 0.95–0.98). In general, the findings of this meta-analysis seem to be similar to our study report with a difference of 0.5 points in sensitivity [11]. In another study conducted by Wu et al. [3] in 2019 on 100 infants with cholestasis (36 infants with biliary atresia) with a mean age of 43 days. The findings of this study showed that consistent with our results, serum levels of MMP-7 are significantly higher in neonates with BA than in non-BA neonates. The results also showed that the serum level of MMP-7 above 1.43 ng / ml was a predictor of biliary atresia in neonates with cholestasis (diagnostic accuracy, 88%) [3]. Recently, a retrospective study in Japan of individuals under 6 months of age showed that serum concentrations of MMP-7 were higher in BA at diagnosis (median, 89.1 ng / ml) than in non-BA (11.0) or healthy neonates (10.3) were significantly higher [19]. In addition, the area under the ROC curve for MMP-7 in BA versus non-BA subjects was 0.99 (95% confidence interval, 0.96–1.00). However, the sensitivity and specificity at the optimal cut-off of 18.6 ng / ml for MMP-7 serum in the diagnosis of BA were 100 and 90%, respectively [19].

As in the study of Jing et al. the results showed that the level of MMP7 in the BA group was much higher than other causes of cholestasis [20]. In our study, this difference was also significant, but the mean levels of MMP7 in the mentioned study for BA was 38.89 and in our study is estimated at 15.91. Also, in the mentioned study, the level of MMP7 for other causes of cholestasis was 4.4 ng/ml, but in our study it had an average of 4.73. In the mentioned study the AUC was 0.9829 for MMP-7 and the sensitivity, specificity, positive predictive value and negative predictive value were 95.19%, 93.07%, 97.27%, and 91.43%, respectively, which indicates a sensitivity almost identical to that reported in our study, which was 95.5. Furthermore, the cut off value for differentiating BA from other causes of cholestasis was 10.37 ng/ml, while in our study this cut-off point was 7.8 for MMP7 and 434.5 for GGT. Also, in our study AUC for the two markers MMP7 and GGT were 0.988 ± 0.008 and 0.854 ± 0.052, respectively. As reported in the Mizuochi study [21], based on the results of his colleagues, MMP7 had a higher sensitivity and specificity to GGT for the diagnosis of BA, which the findings of our study also confirmed this result. Also, the results of the study of Hirfanglou et al. [22] on the population of healthy infants and neonates in terms of GGT level showed that GGT levels in male infants were higher than female and in vaginal delivery higher than cesarean section and also in preterm infants more than term and finally in the first week was more than other weeks of neonates and in neonates more than other periods of infancy [22], in our study, the level of GGT in the healthy group in boys was significantly higher.

Differences in results and the sensitivity and specificity observed for the biomarkers examined in the studies can be due to several reasons. For example, it has been suggested that serum levels reported for MMP-7 may vary based on factors such as ethnicity, patient age, and type of biomarker kit used [3]. Serum levels of this biomarker have been reported to be significantly lower in people with BA who have undergone cholestatic testing at a younger age [3]. Therefore, the difference in the results of different studies may be due to the age difference of the participants in the study. It has also been shown that the type of kits used to measure biomarkers can produce different results [23]. So that some kits show different sensitivity to factors such as sample size and time. Therefore, it is suggested that different institutes do more research to validate the diversity between these kits before using them in clinical practice. In addition, evidence has reported that serum MMP-7 samples may be degraded during the sample collection period, and their degradation over time may be another difference between the results of this study and other studies. However, according to recent reports, the degradation of MMP-7 over time does not appear to be a potential cofounding factor.

Biliary atresia, a progressive fibro-inflammatory disease of the intrahepatic and extrahepatic biliary tree, is the most common clinical indication for pediatric liver transplantation globally [10, 14]. Therefore, a good and accurate diagnostic tool in these patients is essential for earlier diagnosis and prevention of liver transplantation. Hence, other noninvasive biomarkers of liver fibrosis remains needed. Examining the clinical features of neonatal cholestasis facilitates the decision to perform the best invasive procedure (such as liver biopsy and intraoperative cholangiography) to diagnose BA and improve outcomes. MMP-7 was a primary pathogenic mediator in the evolution of fibrotic lesions by degrading E-cadherin in kidney fibrosis [24]. Chatmanee et al. found that preoperative serum MMP7 levels were correlated with inflammation classifications [25]. Jiang et al. reported that the preoperative serum MMP-7 level was correlated with the fibrosis stage in liver biopsies, whereas no significance was found within different inflammation grades [20]. Suggested that MMP-7 was produced by severe bile duct inflammation and promoted liver fibrosis through the E-cadherin/β- catenin pathway [26]. MMP-7 could reflect the early stage of bile duct injury before the bile duct obstruction develops [4, 14, 20]. While other indicators (TBIL, DBIL, GGT, APRI, Fibro touch) could only reflect the result of bile duct obstruction and liver fibrosis [4, 14, 20]. Moreover, simultaneous observation of serum and stool MMP-7 level could monitor the degree of inflammation, obstruction of the extrahepatic bile duct, and liver fibrosis more objectively than stool color [5, 8]. Similar to MMP-7’s early diagnostic ability, this evidence suggested that MMP-7 could make better early predictions of survival with native liver comparing to other prognostic factors [7].

Due to the vulnerability of GGT to other conditions such as gender, age, etc., one of the reasons for the preference of MMP7 is that this biomarker is less affected by other variables. It is suggested that a study be conducted with the purpose of MMP7 levels and its relationship with ultrasound and liver biopsy findings and changes in serum levels of this biomarker after liver transplantation.

### Limitation

Because this study was performed in Tertiary hospitals and patients with cholestasis received previous workouts and sometimes various treatments before visiting this center, limited access to their medical records and the impossibility of eliminating the effect of previous medications on the result of this study, it seems to be the main limitation of this study.

## Conclusion

Due to the higher specificity and sensitivity of MMP7 than GGT, this biomarker demonstrated good accuracy to differentiate biliary atresia from other causes of cholestasis. However, prospective studies with higher sample sizes are recommended to examine these biomarkers, especially considering potential and effective confounders in this age group.

## Data Availability

Data is available upon request from the corresponding author for the article due to privacy / ethical restrictions.
